# Progress in Cerebral Surgery

**Published:** 1903-08-01

**Authors:** 


### Progress in Cerebral Surgery.

Facial Paralysis.—A considerable number of cases of facial paralysis resulting from middle ear disease tend to recover or very greatly improve; cases resulting from trauma, such as bullet wounds, fracture of the skull, or division in mastoid operations do not recover, and the distressing condition remains permanent. For the relief of this condition some recent work has been done, the results of which are so far encouraging, but sufficient time has not yet elapsed to arrive at conclusions as to the ultimate results or possibilities. C. A. and H. A. Ballance in conjunction with Purves Stewart[1] have communicated the results of seven cases of facial paralysis treated by nerve anastomosis; in six of these the divided facial was united to the spinal accessory, in the seventh case to the hypoglossal. The first case was operated upon by Ballance in 1895 for paralysis after a mastoid operation. The patient was not seen again for seven years, when he reappeared in 1902. His condition then was one of left facial paralysis flaccid in type, the muscles of the face all reacted briskly to faradism and galvanism, taste in the anterior part of the tongue was still absent, no dissociated movements of the face were possible, but on movement of the left shoulder against resistance marked contractions occurred on the left side of the face. These operations were performed at periods of from five months to three years after the onset of paralysis, the interval generally selected being six months. If the paralysis after injury to the facial nerve persists without improvement for six months natural recovery is probably hopeless. The method of anastomosis adopted is to unite the stump of the divided facial nerve, either end to end to the cut trapezius branch of the spinal accessory, or else to make an end-to-side anastomosis by splitting the sheath of the spinal accessory. A microphotograph is shown of a section made 13 months after facio-spinal anastomosis in a dog. The end-to-side method was adopted, and the preparation demonstrates well the down growth of new fibres from the spinal accessory into the peripheral portion of the facial. Within periods varying from three to nine months movements began to appear in the facial muscles, but always in association with movements of the shoulder, attempts to move the shoulder producing contraction of the facial muscles; in none of the cases, so far, have voluntary dissociated movements of the face taken place. Temporary paralysis of the sterno-mastoid and trapezius occurs, but clears up completely in all cases. It has been noticed that, where the paralysed facial muscles have undergone contracture and become spastic, very soon after anastomosis they become flaccid. When the muscles have ceased to respond to faradisation through the skin, the reaction to faradisation of the divided facial trunk during the operation produced marked contractions; this the authors consider a clinical proof of regeneration taking place in the peripheral portion of divided nerves.[2] After operation motor power returns to the muscles considerably before faradic excitability. Cushing[3] records a case of facial paralysis following a bullet wound, the nerve was completely divided and paralysis complete. Six weeks afterwards he made an end-to-end anastomosis with the spinal accessory, daily galvanism of the face was continued for several months afterwards. After about three months the patient noticed that when he attempted to raise his shoulder contraction of the facial muscles occurred. The tone of the facial muscles very markedly improved, so that the disfigurement during repose of the face was scarcely noticeable. At the end of nine months volitional control of individual groups of facial muscles had begun to return and could be dissociated to a considerable extent from shoulder movements; when last observed the improvement was continuing, the patient daily practising movements before a glass. The result in this case is more hopeful so far than in any of the others; the patient helped himself very much by daily endeavours to bring out the movements, and by so doing educated his cortical centres to adapt themselves to the new condition of things. Whether the shoulder centre has to cultivate the power of developing facial movements, or whether the facial centre has to endeavour to transmit impulses by new paths we do not know, but in either case the power must come slowly and be attained by a gradual education; especially must this be so if a centre concerned with such coarse movements as affect the shoulder has to be educated to conduct the minute and intricate co-ordinate action of the facial muscles. As an endeavour to meet this difficulty Ballance[4] advocates the anastomosis of the divided facial with the hypoglossal nerve, as the tongue centre is so much more intimately connected with that of the face, and would probably much more readily adapt itself to presiding over facial movements. Moreover, the facial and hypoglossal nuclei in the bulb are in close relationship to each other. In the last case on which he operated he used the hypoglossal, but sufficient time has not yet elapsed to judge of the results. Körte[5] united the divided facial to the hypoglossal in 1901. Some improvement was noted in two months, the patient observing that on moving the tongue the facial muscles moved also. Within twelve months some voluntary dissociated movements of the face were possible. The reaction of degeneration had quite disappeared. This case has now been under observation for over a year, but the result does not seem to be much better than in the recorded cases of facio-spinal anastomosis.

[1] Brit. Med. Jour., May 2. 1903. [2] The Healing of Nerves, C. A. Ballance. 1901, Macmillan and Co. [3] Anns. of Surgery, May 1903. [4] Brit. Med. Jour., May 2, 1903. [5] Lancet, May 23, 1903.

